# Current ventilation practice during general anaesthesia: a prospective audit in Melbourne, Australia

**DOI:** 10.1186/1471-2253-14-85

**Published:** 2014-10-01

**Authors:** Dharshi Karalapillai, Laurence Weinberg, Jonathan Galtieri, Neil Glassford, Glenn Eastwood, Jai Darvall, Jake Geertsema, Ravi Bangia, Jane Fitzgerald, Tuong Phan, Luke OHallaran, Adriano Cocciante, Stuart Watson, David Story, Rinaldo Bellomo

**Affiliations:** Department of Intensive Care, Austin Hospital, Melbourne, Australia; Department of Anaesthesia, Austin Hospital, Melbourne, Australia; Department of Anesthesia, Royal Melbourne Hospital, Melbourne, Australia; Department of Anaesthesia, Northern Hospital, Melbourne, Australia; Department of Anaesthesia, Box Hill Hospital, Melbourne, Australia; Department of Anaesthesia, Alfred Hospital, Melbourne, Australia; Department of Anaesthesia, St Vincents Hospital, Melbourne, Australia; Department of Anaesthesia, Monash Medical Centre, Melbourne, Australia; Department of Anaesthesia, Western Health, Melbourne, Australia; University of Melbourne, Melbourne, Australia; Intensive Care Research, Austin Hospital and Co-director, Australian and New Zealand Intensive Care Research Centre (ANZIC-RC), Melbourne, Australia; Department of Epidemiology and Preventive Medicine, Monash University, Melbourne, Australia

**Keywords:** Tidal volume, PEEP, Intraoperative ventilation, Anaesthesia

## Abstract

**Background:**

Recent evidence suggests that the use of low tidal volume ventilation with the application of positive end-expiratory pressure (PEEP) may benefit patients at risk of respiratory complications during general anaesthesia. However current Australian practice in this area is unknown.

**Methods:**

To describe current practice of intraoperative ventilation with regard to tidal volume and application of PEEP, we performed a multicentre audit in patients undergoing general anaesthesia across eight teaching hospitals in Melbourne, Australia.

**Results:**

We obtained information including demographic characteristics, type of surgery, tidal volume and the use of PEEP in a consecutive cohort of 272 patients. The median age was 56 (IQR 42–69) years; 150 (55%) were male. Most common diagnostic groups were general surgery (31%), orthopaedic surgery (20%) and neurosurgery (9.6%). Mean FiO_2_ was 0.6 (IQR 0.5-0.7). Median tidal volume was 500 ml (IQR 450-550). PEEP was used in 54% of patients with a median value of 5.0 cmH_2_O (IQR 4.0-5.0) and median tidal volume corrected for predicted body weight was 9.5 ml/kg (IQR 8.5-10.4). Median peak inspiratory pressure was 18 cmH_2_O (IQR 15–22). In a cohort of patients considered at risk for respiratory complications, the median tidal volume was still 9.8 ml/kg (IQR 8.6-10.7) and PEEP was applied in 66% of patients with a median value of 5 cmH_2_0 (IQR 4–5). On multivariate analyses positive predictors of tidal volume size included male sex (p < 0.01), height (p = 0.04) and weight (p < 0.001). Positive predictors of the use of PEEP included surgery in a tertiary hospital (OR = 3.11; 95% CI: 1.05 to 9.23) and expected prolonged duration of surgery (OR = 2.47; 95% CI: 1.04 to 5.84).

**Conclusion:**

In mechanically ventilated patients under general anaesthesia, tidal volume was high and PEEP was applied to the majority of patients, but at modest levels. The findings of our study suggest that the control groups of previous randomized controlled trials do not closely reflect the practice of mechanical ventilation in Australia.

## Background

It is estimated that 783,000 patients undergo general anaesthesia per year in Victoria, Australia [1]. In this setting, mechanical ventilation is often mandatory to support respiratory function. Despite its necessity, mechanical ventilation has many potentially detrimental effects on lung tissue. In the context of critical care medicine, ventilator induced lung injury can result from cyclic overstretching of aerated alveoli with high tidal volume ventilation, from repeated opening and closing of peripheral airways, from low lung volume associated with recruitment and de-recruitment of unstable lung units, and from the application of high airway pressures [2, 3]. Whether short exposure to potentially injurious mechanical ventilation in patients with healthy lungs is sufficient to initiate lung damage is a subject of controversy. Currently the optimal management of intraoperative mechanical ventilation is not known. In particular, uncertainty exists with regard to the optimal tidal volume and the need for and the optimal value of positive end expiratory pressure (PEEP).

Randomized trials support the use of a low tidal volume strategy and the application of PEEP in critically ill patients with acute lung injury or acute respiratory distress syndrome (ARDS), who require prolonged mechanical ventilation [4, 5]. These findings suggest that it may be physiologically logical and desirable to apply a similar ventilation strategy to other patient groups such as patients undergoing intra-operative ventilation under general anaesthesia. A recent randomized controlled trial of patients at risk of respiratory complications undergoing major abdominal surgery showed that a low tidal volume strategy and the application of PEEP had clinical advantages over standard care [6]. In the standard care group of this study, the average tidal volume of > 11 ml/kg and PEEP was not used. Furthermore, recently a large multi centre trial in a similar cohort of patients compared a low PEEP versus high PEEP strategy when combined with a tidal volume of 8 ml/kg found no significant difference in the incidence of respiratory complications [7]. The relevance and external validity of these findings in other health care systems remains unclear, as there is no understanding or knowledge of current practice in other developed countries such as Australia. We hypothesized that anaesthetists in Australia would adopt a “conventional strategy” of high tidal volume (i.e. 10–12 ml/kg) with the application of PEEP. Accordingly, we conducted a prospective multicentre audit to define current practice of intra-operative mechanical ventilation in the city of Melbourne, Australia.

## Methods

With ethics approval (HREC/14/Austin/260) and in accordance with the STROBE guidelines for observational studies, we conducted a multicentre audit across eight teaching hospitals in Melbourne, Australia [8]. These included: The Austin Hospital, The Royal Melbourne Hospital, The Northern Hospital, St Vincents Hospital, The Alfred Hospital, Monash Medical Centre, The Western Hospital and Box Hill Hospital. Five of these hospitals were tertiary referral hospitals with individual centres being statewide referral centres for liver transplant, spinal injury, trauma, and cardiac and lung transplantation. One tertiary centre and all secondary centres included obstetric services. We collected data on all adults (greater than 18 years) patients undergoing surgery at two distinct time points (10 h00 and 14 h00), on three consecutive days (28/8/2013 to 30/8/2013). At these designated times, an observer entered the operating room and obtained information regarding demographic characteristics, patient co-morbidities, type and predicted length of surgery and details of intra-operative ventilation including the patient-ventilator interface used, inspired oxygen concentration, ventilation mode, tidal volume, respiratory rate, use and amount of PEEP applied, and peak inspiratory pressures. Treating anaesthetists were blinded to the purpose of the study. Predicted body weight was calculated as 50 + 2.3 [height(cm)/(2.54- 60)] for men and 45.5 + 2.3 [height (cm)/(2.54- 60)] for women [9].

We analysed the following subgroups i) patients who underwent general anaesthesia; ii) patients whose ventilator interface was an endotracheal tube; and iii) patients whose ventilator interface was an endotracheal tube and who were considered at increased risk of respiratory complications on the basis of age, type and duration of surgery, emergency surgery and the presence of comorbid respiratory disease or recent respiratory infection [10, 11]. We excluded patients who underwent intracranial surgery from this high-risk group due to concerns about intracranial hypertension potentially influencing ventilator settings (i.e. a low tidal volume strategy may lead to hypoventilation, hypercapnia and increased intracranial pressure).

### Data handling and statistical analysis

Data were incomplete for 16.5% (n = 33) of outcomes. Outcomes of interest included use of PEEP (of any value) and tidal volume selected. Descriptive statistics were tabulated for all patients receiving general anaesthesia, by method of airway management, by hospital type, by individual site, and by high risk of respiratory complications. Univariate comparisons of means, medians or proportions, as appropriate, were performed. Factors thought to be clinically important in determining the application of PEEP were considered for multi-variable logistic regression analysis. Only patients undergoing general anaesthesia requiring endotracheal intubation and mechanical ventilation were considered. Model variables considered included: age, sex, ASA score, presence of significant respiratory disease, type of surgery (e.g. emergency, general, cardiothoracic, neurosurgery), prolonged duration of surgery (>2 hours), type of hospital (secondary or tertiary centre), height and weight. To assess the robustness of the model, height and weight were replaced with body mass index, and predicted lean body weight. The area under the receiver operator characteristic curve (c-statistic, AuROC) and the Hosmer-Lemeshow (goodness-of-fit) test were used to assess model adequacy. Data are presented as odds ratios (OR) with 95% confidence intervals (CI). A p-value < 0.05 was considered statistically significant.

To identify independent predictors of intra-operative tidal volume in intubated patients undergoing general anaesthesia, backwards multiple linear regression analysis was performed using those factors thought to be clinically relevant as independent covariates: height, weight, age, ASA score, gender, presence of significant respiratory disease, type of surgery (emergency, cardiothoracic, major abdominal, neurosurgery), prolonged duration of surgery (>2 hours), and type of hospital (secondary or tertiary centre). Data are presented as estimates with standard errors, and β standardized coefficients. A p-value < 0.05 was considered statistically significant. SPSS version 20 (IBM, North Castle, NY, USA) was used for all statistical analyses.

## Results

### Demographics

In a cohort of 272 consecutive patients from eight teaching hospitals in the city of Melbourne, Australia, the median age was 56 years (IQR 42–69) and 150 (55%) patients were male. The most common types of surgery performed were general in 86 (31.6%) patients, orthopaedic in 55 (20.2%) patients and urological in 20 (7.4%) patients. Overall, 26 (9.6%) patients underwent intracranial neurosurgery. Patient characteristics are summarized in Table 1).

**Table 1:** **Characteristics of study patients**

Age in years	56 (42–69)
Male (%)	150 (55)
Height in cm	170 (162–176)
Actual weight in kg	75 (67–89)
Ideal body weight in kg	52.9 (IQR 48.6-58.6)
Body mass index in kg per m^2^	25.9 (IQR 24.4- 30.8)
**ASA status (%)**	
I	48 (17.6)
II	107 (39.4)
III	81 (29.8)
IV	29 (10.6)
V	1 (0.4)
Unknown	6 (2.2)
Emergency	22 (8.1)
Respiratory disease (%)	73 (26.8)
Cardiac disease (%)	66 (24.3)
**Type of surgery (%)**	
General	86 (31.6)
Cardiac	21 (7.7)
Thoracic	6 (2.2)
Maxillofacial/Dental	9 (3.3)
Gynaecology	2 (0.7)
Obstetrics	4 (1.4)
Opthalmology	4 (1.5)
Orthopaedics/Spinal	55 (20.2)
Neurosurgery	26 (9.6)
Urology	20 (7.4)
Vascular	15 (5.5)
Plastics/Burns	16 (6.1)
Other	8 (3)
**Hospital type (%)**	
Secondary	48 (17.6)
Tertiary	224 (82.4)
**Type of anaesthesia (%)**	
General anesthesia	262 (96.3)
Regional anaesthesia	10 (3.7)
**Expected duration of surgery (%)**	
<30 mins	12 (4.4)
30-60 mins	56 (20.6)
1-2 hrs	82 (30.1)
2-4 hrs	79 (29.0)
>4 hrs	30 (11.0)
Unknown	13 (4.9)

Values are median (IQR) or number (percentage).

For the 262 patients undergoing general anaesthesia, an endotracheal tube was used in 200 (76%) patients and a supraglottic airway device in 41 (16%) patients with 17 (5%) of patients receiving facemask anaesthesia. One patient (0.4%) had a tracheostomy in situ and 1 (0.4%) patient underwent rigid bronchoscopy. For the 200 patients with an endotracheal tube, volume control mode was used in 146 (73%) patients, while 46 (23%) patients received pressure control ventilation and only 2 patients received pressure support ventilation. Mean Fi02 was 0.6 (IQR 0.5-0.7) and median absolute tidal volume was 500 ml (IQR 468–571). Median tidal volume per predicted body weight was 9.7 ml/kg (IQR 8.6-10.5). PEEP was used in 132 (66%) patients with a median value of 5 cmH_2_0 (IQR 4–5). Median maximum inspiratory pressure was 19 cmH_2_O (IQR 16–22). Details of ventilation during anaesthesia are summarized in Table 2. For the 81 patients who were considered at increased risk of perioperative respiratory complications, the median tidal volume was 9.8 ml/kg (IQR 8.6-10.7) lean body mass and PEEP was applied in 68% of cases at a median level of 5 cmH_2_O (IQR 4–5).

**Table 2:** **Details of ventilation during anaesthesia**

	All patients N = 272	GA N = 262	GAETT =200	GA ETT High risk N = 81
**Height (cm)**	170 (162–176)	170 (162–176)	170 (162–175)	170 (162–175)
**Absolute weight (kg)**	75 (67–89)	75 (67–88)	78 (67–90)	79 (65–89)
**Ideal body weight (kg)**	52.9 (48.6-58.6)	52.9 (48.6-58.6)	53.8 (48.4-58.7)	54.3 (48.4-58.5)
**Body mass index (kg/m** ^**2**^ **)**	26 (24.4-30.8)	25.9 (23.4-30.0)	26.2 (23.8-31.2)	26.3 (23.4-31.1)
**Patient-ventilator interface (%)**				
ETT	200 (73.5)	200 (76.3)	200 (100)	81 (100)
LMA	41 (15.1)	41 (15.6)	0	0
Mask	17 (6.3)	17 (4.5)	0	0
Tracheostomy	1 (0.4)	1 (0.4)	0	0
Nil (including rigid bronchoscopy)	11 (4.1)	1 (0.4)	0	0
Unknown	2 (0.7)	2 (0.8)	0	0
**FiO**_**2**_	0.60 (0.5-0.7)	0.60 (0.50-0.70)	0.60 (0.50-0.70)	0.60 (0.50-0.75)
**Ventilation mode (%)**				
VCV	148 (54.4)	148 (48.9)	146 (73)	59 (72.8)
PCV	54 (19.4)	54 (20.6)	46 (23)	17 (21)
PSV	10 (3.7)	10 (3.8)	2 (1)	2 (2.5)
SV	47 (17.3)	37 (14.1)	0	0
Jet	1 (0.4)	1 (0.4)	0	0
Unknown	12 (4.8)	12 (4.6)	6 (3)	3 (3.7)
**Absolute tidal volume (ml)**	500 (450–550)	500 (450–550)	500 (468–471)	500 (475–578)
**Tidal Volume (ml/kg lean body mass)**	9.5 (8.5-10.4)	9.5 (8.5-10.4)	9.7 (8.6-10.5)	9.8 (8.6-10.7)
**Respiratory Rate (b/minute)**	12 (10–12)	12 (10–12)	12 (10–12)	11 (10–12)
**PEEP Yes (%)**	146 (53.7)	146 (55.7)	132 (66 %)	51 (63)
**PEEP (cmH**_**2**_ **0)**	5.0 (4.0-5.0)	5.0 (4.0-5.0)	5.0 (4.0-5.0)	5.0 (4.0-5.0)
**Peak inspiratory pressure (cmH**_**2**_ **0)**	18 (15–22)	18 (15–22)	19 (16–22)	19 (16–22)

Values are median (IQR) or number (percentage); *GA* general anaesthesia, *GAETT* general anaesthesia with an endotracheal tube, *GAETT* high risk: general anaesthesia with an endotracheal tube with increased risk of respiratory complications, *FiO*_*2*_ inspired oxygen concentration, *ETT* endotracheal tube, *LMA* laryngeal mask airway, *VCV* volume control ventilation, *PCV* pressure control ventilation, *PSV* pressure support ventilation, *SV* spontaneous ventilation, *PEEP* positive end-expiratory pressure.

In tertiary hospitals the median tidal volume per predicted body weight was 9.7 ml/kg (IQR 8.9-10.8) and 120 of the 176 (66.3%) patients received PEEP at a median value of 5 (IQR 4–5). Results were similar in secondary hospitals with a median tidal volume of 9.9 ml/kg (IQR 7.9-12.5) and 22 of 34 (64.7%) patients had PEEP applied at a median value of 5 cmH20 (IQR-5-5.25).

### Predictors of intra-operative PEEP use and tidal volume

On multi-variable logistic regression analysis, type of hospital (OR 3.11; 95% CI: 1.05 to 9.23, p = 0.04) and prolonged surgery (OR 2.47; 95% CI: 1.04 to 5.84, p = 0.04) were positive independent predictors of PEEP use, while cardiothoracic surgery and neurosurgery were negative independent predictors of the use of intra-operative PEEP (Table 3). These relationships remained unchanged when BMI and predicted body weight were substituted for absolute weight and height in the model. Height, weight and male gender were positive predictors of higher tidal volume; ASA score was a negative predictor of higher tidal volume (Table 4).

**Table 3:** **Multi-variable logistic regression analysis for use of intra-operative PEEP in intubated patients undergoing general anaesthesia**

Received PEEP	OR	95% CI	p-value
Tertiary centre	3.11	1.05 - 9.23	0.04
Type of surgery			
Cardiothoracic	0.19	0.05 - 0.70	0.01
Neurosurgery	0.26	0.08-0.91	0.04
Prolonged surgery (>2 hours)	2.47	1.04 - 5.84	0.04

AUC 0.76 (95% CI: 0.69 to 0.84); Hosmer-Lemeshow GoF, p = 0.84.

(*OR* Odds Ratio, *CI* Confidence Interval).

**Table 4:** **Multiple linear regression analysis for use of intra-operative tidal volume in intubated patients undergoing general anaesthesia**

Variable	Estimates	Standard error	95% CI	β-co-efficient	p-value
Male	49.17	12.66	24.16 to 74.17	0.29	<0.001
ASA score	−23.57	9.79	−42.9 to −4.25	−0.16	0.02
Weight	1.15	0.28	0.60 to 1.71	0.28	<0.001
Height	1.38	0.67	0.06 to 2.7	0.16	0.04

(95% CI: 95% confidence interval of the estimate; β-co-efficient: standardised estimate expressed as standard deviations).

## Discussion

### Key findings

We conducted a prospective audit in eight teaching hospitals in the city of Melbourne, Australia to determine current ventilation practice during anaesthesia. We found that, in mechanically ventilated patients under general anaesthesia, tidal volume was high (approximately 10 ml/kg predicted body weight) and that PEEP was applied to the majority of patients, but only at modest levels (approximately 5 cmH_2_O). In addition, we found that the type of hospital and prolonged surgery were both positive independent predictors of PEEP use, whilst cardiothoracic surgery and neurosurgery were negative independent predictors for the use of PEEP. Significant predictors of higher tidal volume were gender, height, and weight. Furthermore, even in patients at high risk of respiratory complications, the average tidal volume was approximately 10 ml/kg lean body mass and there was no difference in the use of PEEP.

### Comparison with previous studies

To our knowledge, this the first multicentre study evaluating intra-operative ventilation practices in Australian teaching hospitals. A recent retrospective analysis of over 200,000 patients from two institutions in the United States reported similar findings with a median tidal volume of around 9 ml/kg and PEEP use in approximately 60% at a median amount of 5 cmH_2_0 [12]. Our findings are also consistent with a subsequent retrospective study of over 29,000 patients from a single centre in the United States who reported a similar median tidal volume [13].

However, in a multicentre study of a mixed surgical population in France, the frequency of PEEP use was only 19% [14]. Predictors of the use of PEEP in our study included surgery in a tertiary hospital and expected prolonged duration of surgery. The former suggests that the use of PEEP may be institution specific. Interestingly, increased weight did not predict the use of PEEP, a finding that contrasts with other studies [13]. Predictors of the absence of PEEP in our study included patients undergoing cardiothoracic surgery and neurosurgery. The omission of PEEP in patients undergoing neurosurgery is logically explained on the basis of a potential exacerbation of intracranial hypertension with PEEP. However its omission from cardiothoracic surgery is of interest given this group is considered at increased risk of peri-operative respiratory complications [15]. One possible explanation for this relationship may be that many of our observations in cardiac surgery patients may have been taken when ventilation was suspended whilst patients were on cardiopulmonary bypass. It is a common practice for PEEP to be removed at this time to facilitate surgical access.

**Figure 1:**
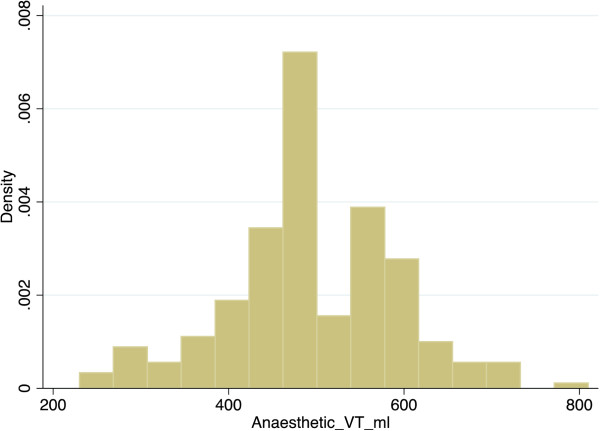
**Histogram of the distribution of tidal volume expressed as density of patients who underwent general anaesthesia with an endotracheal tube (VT = tidal volume, ml = millilitres).**

The median tidal volume in our study was 500 ml, which was also the most frequent tidal volume used (Figure 1). This may be explained by the default setting for tidal volume in volume control mode on many anaesthesia machine ventilators being 500 ml. In our study the positive predictors of increased tidal volume included male gender, height and weight. The association of the latter two with tidal volume is similar to published findings [14]. Male gender has been previously associated with lower tidal volumes [14]. However in our study a higher tidal volume was delivered to males, which was independent of their actual weight and height. This suggests that anaesthetists may have set tidal volumes with an assumption of a higher body weight in these patients, irrespective of their actual measured weight. One potential explanation of the high tidal volume approach to ventilation in our study may be that Australian anaesthetists calculated tidal volume according to absolute body weight rather than predicted body weight, even though predicted body weight has been used to calculate tidal volume in previous studies of intra-operative ventilation [4–6, 10, 16–19].

Despite the necessity and frequency of intra-operative mandatory ventilation, the optimal ventilator settings with regard to tidal volume and PEEP are not known. Previous recommendations have advocated a high tidal volume strategy of 10–12 ml/kg predicted body weight, citing reduced oxygen requirements and reduced incidence of atelectasis as potential benefits [20]. It appears that our findings with respect to tidal volume are more consistent with a traditional approach (10 ml/kg) as opposed to a low tidal volume strategy (6 ml/kg).

The benefits of a low tidal volume strategy first became apparent in randomized controlled trials of critically ill patients with acute respiratory distress syndrome [19–21]. A lung protective strategy (low tidal volume and the application of PEEP) resulted in reduced mortality and is now considered standard of care in these patients. However, studies of a low tidal volume strategy in patients without lung injury when mechanically ventilated under anaesthesia have yielded inconsistent results [10, 15, 22, 23]. On the one hand, low tidal volume strategies have been associated with reduced lung and systemic inflammatory responses [24–26] and reduced respiratory complications [27]. Moreover, experimental and clinical studies have suggested that mechanical ventilation using large tidal volumes could initiate lung injury in healthy lungs [10, 28–34] especially during major surgery with its associated inflammatory response, which makes the lungs more vulnerable to mechanical ventilation induced injury [35–38]. On the other hand, many of these studies of low tidal volume have used small patient populations or have focused on different and not necessarily clinically relevant outcomes [23]. This has led to significant controversy in the literature with some authors suggesting that a low tidal volume strategy is unnecessary in patients without acute lung injury, citing the need for increased oxygen requirement, increased risk of auto-PEEP (due to high respiratory rates), hypercapnia and atelectasis [22] as potentially harmful effects [34]. In this regard, a retrospective analysis of over 29,000 patients suggested the use of low intraoperative tidal volume with minimal PEEP (4 cmH20) is associated with an increased 30-day mortality when compared to a more conventional ventilation strategy with minimal PEEP [13]. The authors speculated that this might have been related to an increased atelectasis. Experimental evidence has linked this with increased bacterial growth and translocation and the development of lower respiratory tract infections [39, 40].

Recently, in a multicenter randomized trial, Futier et al. investigated the effect of a low tidal volume strategy in patients undergoing major abdominal surgery who were considered at increased risk for perioperative respiratory complications [6]. Their findings suggested that a low tidal volume strategy with PEEP was associated with reduced need for postoperative ventilator assistance. However, the standard care group included a ventilation strategy with an average tidal volume in excess of 11 ml/kg and no PEEP [6]. Similarly in a study performed by Servegnini and colleagues in major abdominal surgery [27], a tidal volume of 7 ml/kg and PEEP 10 cmH_2_0 in conjunction with recruitment manoeuvres was associated with improved postoperative pulmonary function tests, gas exchange, chest x-ray findings and reduced pulmonary infections when compared to a standard care group who received a tidal volume of 9 ml/kg and no PEEP or recruitment manoeuvres. However the absence of PEEP in the standard care arm of both of these studies is not consistent with the Australian cohort of this study. Previous evidence suggests that application of PEEP in itself may be lung protective independent of tidal volumes. PEEP may prevent atelectasis [41], micro-aspiration [42], and decrease the need for rescue therapies for hypoxia [19]. Low or absent PEEP may increase shear stress and lung injury independent of the absence of high plateau pressures due to the cyclic collapse and tidal recruitment of lung units referred to as “atelectatrauma” [43]. Previous work suggests that the combination of a large tidal volume without PEEP may itself induce injury in healthy lungs in proportion to the duration of ventilation and promote pulmonary complications [24].

A recent multicenter study by the PROVE Network investigators directly compared the effect of a low (<2 cmH_2_0) PEEP strategy with a high PEEP strategy (12 cmH_2_0) in patients undergoing major abdominal surgery [7]. A set tidal volume of approximately 8 ml/kg was used in both groups. A high PEEP strategy did not reduce respiratory complications. The authors suggested a lack of efficacy for the prevention of respiratory complications with high PEEP when combined with a low tidal volume strategy. However in this study a low tidal volume of 6 ml/kg as described in previous trials of protective ventilation in acute lung injury and in previous intraoperative studies was not used [4–6].

### Implications for clinical practice

Given our finding of the frequent intraoperative use of PEEP and its divergence from the standard care group of the above randomized controlled trial by Futier et al. [6], caution is required in the interpretation of such a study and in its widespread application worldwide. It is possible that the absence of PEEP in the standard care group may have been the critical variable. In the randomized multicenter trial by the PROVE Network investigators [7], the study group was consistent with our findings with respect to the use of PEEP. However the amount of PEEP used was either significantly lower or significantly higher than our findings. Currently no significant differences in either postoperative lung function or clinical outcomes have been demonstrated between a high and a low tidal volume strategy in patients undergoing major abdominal surgery where both regimens included the application of PEEP [22]. Thus, we suggest that more studies are needed to understand whether a low tidal volume approach is beneficial even in the presence of PEEP and to assess why, even after exclusion of neurosurgery, in close to one fourth of patients, PEEP is not applied.

### Strengths and limitations

Our study is the first to assess current practice of intraoperative ventilation in Australian hospitals. It is prospective and multicenter in nature and included data from a representative sample of over 270 patients from eight teaching hospitals. Whilst our study was performed in a single city, our data included many anesthetists from multiple hospitals and we think it is likely to be representative of national practice in Australia. Treating anaesthetists were blinded to the purpose of the study and only the site investigator and, in some cases, the research coordinator at specific sites were aware of the conduct of the study.

A potential limitation of our study is that it did not exclusively include patients who would be considered at increased risk for postoperative complications. In addition the types of surgery were diverse. However, we wanted to study current practice across all types of patients and a subgroup analysis of patients at increased risk for postoperative acute lung injury found that ventilator practice was the same. We also performed an analysis excluding patients who may have had PEEP or a low tidal volume strategy excluded for other reasons (i.e. intra-cranial neurosurgery). Despite this, the results remained similar. Our study only evaluated ventilator settings at one time point intra-operatively and this observation was assumed to represent the ventilator settings for the duration of the surgery. Potentially ventilator settings may have been altered over the course of the procedure according to clinical need after our data was collected.

## Conclusions

In our cohort of mechanically ventilated patients under general anaesthesia, tidal volume was high and PEEP was applied to the majority of patients but at modest levels. Our findings suggest that, in an Australian cohort, a liberal tidal volume strategy in combination with modest PEEP in selected patients is likely the most common strategy for intra-operative mechanical ventilation and that PEEP application is the dominant standard of care. The findings of our study suggest that the control groups of previous randomized controlled trials do not closely reflect the practice of mechanical ventilation in Australia. In this regard we suggest future clinical research should compare “low tidal volume ventilation with PEEP” to “standard volume ventilation with PEEP”. This would allow direct comparison of a low tidal volume ventilator strategy to the current standard of care ventilation practices we observed in this prospective audit of ventilation practices during general anaesthesia in Australian operating rooms.

## Abbreviations

PEEP: Positive end expiratory pressure
FiO_2_: Inspired oxygen concentration
ICU: Intensive care unit
ARDS: Acute respiratory distress syndrome
cm: Centimetres
ASA: American Society of Anesthesiologists
AuROC: Area under receiver operator curve
OR: Odds ratio
CI: Confidence interval
IQR: Interquartile range
BMI: Body mass index
GA: General anaesthesia
GAETT: General anaesthesia with an endotracheal tube
GAETT high risk: General anaesthesia with an endotracheal tube with increased risk of respiratory complications
ETT: Endotracheal tube
LMA: Laryngeal mask airway
RB: Rigid bronchoscopy
VCV: Volume control ventilation
PCV: Pressure control ventilation
PSV: Pressure support ventilation
SV: Spontaneous ventilation.
